# Structural Characteristics of Insoluble Dietary Fiber from Okara with Different Particle Sizes and Their Prebiotic Effects in Rats Fed High-Fat Diet

**DOI:** 10.3390/foods11091298

**Published:** 2022-04-29

**Authors:** Hongliang Fan, Ying Zhang, Mohammed Sharif Swallah, Sainan Wang, Jiarui Zhang, Jiaqi Fang, Jiahong Lu, Hansong Yu

**Affiliations:** 1 College of Food Science and Engineering, Jilin Agricultural University, Changchun 130118, China; f_hongliang@163.com (H.F.); zhang577726644@163.com (Y.Z.); 18644941109@163.com (S.W.); jiarui197@163.com (J.Z.); fjq2825516331@163.com (J.F.); lujiahong1222@163.com (J.L.); 2 National Soybean Industry Technology System Processing Laboratory, Changchun 130118, China; 3 Science Island Branch of Graduate School, University of Science and Technology of China, Hefei 230026, China; m.s.swallah@gmail.com

**Keywords:** okara, insoluble dietary fiber, prebiotic, gut microbiota

## Abstract

Dietary fiber, which is utilized to make functional meals, is an important component for promoting human health and managing calorie consumption. In this study, three different particle sizes of OIDF (Okara insoluble dietary fiber) were characterized. Their lipid-lowering effects and the impacts on gut microbiota were determined by OIDF intervention in high-fat diet rats. Scanning electron microscopy (SEM) results showed that the three particle sizes of OIDF have different morphologies. Fourier transform infrared spectroscopy (FT-IR) results showed that the three sources of IDF samples have similar active groups, but the thermogravimetric analysis/differential scanning calorimetry (TGA/DSC) and X-ray diffraction (XRD) showed that three different particle sizes of OIDF have different retention and crystallinity. Among the three OIDFs, OIDF-10 exhibited the stronger WSC, OHC, CAC, and SCAC. The results after the feeding showed that the OIDF of three particle sizes could improve the elevation of blood lipids and the disturbance of gut microbiota caused by the high-fat diet. Therefore, this study demonstrated the functional significance of the three particle sizes of OIDF and provided a reference for its application in functional food processing, aiming at maintaining healthy blood lipid and intestinal flora levels.

## 1. Introduction

Okara is a by-product of soybean processing. Over 20 million tons of wet soybean dregs (okara) are produced each year in China [1], which is one of the world-leading producers and consumers of soybean. This huge sum of okara produced during soybean processing is evidenced to be packed with a significant amount of nutritional and non-nutritional constituents. However, these are usually used as animal feeds, fertilizers, landfills, or discarded as waste due to their high susceptibility to spoilage, which exerts serious economic and socio-environmental problems [2]. Hence, its valorization will be important to help utilize the untapped nutrients and minimize the environmental problems caused by this waste disposal. Okara is high in nutrients, with insoluble fiber fraction accounting for 70% of the total dietary fiber [1]. Besides, the functional characteristics of insoluble dietary fiber have recently been discovered and gained recognition. Dietary fiber is tagged as “The Seventh Nutrient” [3], and is necessary for a balanced diet [4]. Hence, can be utilized in shaping the gut microbiota [5]. The consumption of a high dietary fiber diet is evidenced by decreases in the bioavailability of some important nutritional components, such as some vitamins and minerals [6], and may also impact the rate of digestion of food substances as well as energy metabolism. The intake of a high-fiber diet can also improve the activities of the gut microbial composition [7].

The gut ecosystem is a home for over 10^14^ microbial cells, and collectively comprise of at least 150 times more genes than their host [8]. In both humans and animals, the gastrointestinal tract is host to a complex community of varied microorganisms, whose activities considerably impact host nutrition and health [9]. The composition of the gut microbiota is said to vary based on genetic characteristics, sex, age, and diet [10]. The association between the host gut-microbiota are dynamic and are highly susceptible to several environmental conditions, mainly diet [8], and the interactive relationship between prebiotics and dietary fiber is reliant on variations in the gut microflora as well as their colonic degradation [7]. Numerous studies indicates that the gut microorganisms can directly impact the physiological conditions of the host organism by encouraging the immune system through stimulating the defensive mechanisms against pathogens and inflammatory bowel diseases, as well as improving the roles of the intestinal barrier, regulating autoimmunity, producing biological metabolites, destroying cancer cells, regulating diabetes and preventing obesity. The host gut-microbiota interactions are highly influenced by several environmental conditions, buy mainly by diet [3,10]. Dietary fiber intake is reported to impact gastrointestinal health via encouraging the gastrointestinal barrier function, nutrient absorption, as well as selectively stimulating the composition and activities of the gut microbes, that can confer health benefits to the host, including lowering the risk of cardiovascular disease, cancer, diabetes, and other ailments [11,12]. For instance, dietary fiber alters the gastrointestinal barrier function through increasing cells and the mucins that produce the “goblet cells” [11]. Mucins are large glycoproteins that, along with antibodies, bacteria, lipids, proteins, ions, antimicrobial peptides, and water, form what is known as mucus. Mucus shields the gut epithelium from mechanical stress, to inhibit the translocation of toxic substances and to lubricate the intestine as well as encourage smooth transportation of digested material [3,12]. In addition, dietary fiber may absorb and excrete toxic chemicals in the intestines, promote intestinal flora, and offer energy and nutrients for probiotic growth [13]. The majority of studies on okara’s insoluble dietary fiber focus on changing their fundamental physical and chemical characteristics [14,15], and only a few studies have been conducted on the functional qualities of okara due to their unpleasant taste [16]. The prolonged intake of a high-fat diet poses a major hazard to human/animal health. Emerging evidence has indicated that high-fat-diet can alter the composition of the microbiota, by inducing dysbiosis or imbalance of the gut microbial ecosystem. Dysbiosis is associated with diseases and metabolic health ranging from insulin resistance, glucose intolerance and to metabolic syndrome [17]. Furthermore, the gut microbiota can influence lipid metabolism via short-chain fatty acid (SCFA) metabolites [18]. SCFAs are primarily generated in the large intestine by anaerobic bacteria fermenting indigestible carbohydrates. They provide the material foundation for the gut flora’s “conversation” with the host [13]. They have the ability to stimulate cell development, enhance intestinal function, and have an impact on cardiovascular metabolism, as well as anti-inflammatory, anti-tumor, and immunological regulatory activities [19].

In this work, high-pressure microfluidic technology was employed to extract three insoluble dietary fibers (IDFs) with varying particle sizes from okara (purity > 90%) to investigate the effects of insoluble dietary fiber from okara (OIDF) on blood lipid levels and intestinal flora in rats fed a high-fat diet. Okara can obtain high-purity OIDF and increase its value addition.

## 2. Materials and Methods

### 2.1. Preparation of OIDFs with Three Different Particle Sizes

Okara, a by-product of producing soybean protein isolate with a protein level of 10–15% (Liaocheng, China), was generously provided by Shandong Jiahua Health Care Products Co., Ltd. The IDF from okara was further processed into three different particle sizes using High Pressure Microfluidic Technology to obtain the OIDFs. The steps comprise of α-amylase treatment at 95 °C for 35 min, being starch glucosidase treated at 60 °C for 30 min, being neutral protease treated 60 °C for 30 min, 70 °C water precipitation for 1.5 h, centrifugation at 3500 rpm for 10 min, alcohol precipitation until colorless, and being freeze-dried for standby. According to the Chinese national standard GB 5009.88-2014, OIDF has a purity of 90.50 percent [20]. The OIDF (purity higher than 90.50%) extracted by biological enzymatic method was processed by dynamic high pressure microfluidization (DHPM). OIDF weighing 20 g was mixed with 800 mL of deionized water, and treated at 10 MPa, 50 MPa, and 150 MPa for 2 min, respectively. Three distinct particle sizes of OIDFs were named OIDF-10 (µm), OIDF-50 (µm), and OIDF-100 (µm).

### 2.2. Determination of the Basic Components of the Three OIDFs

The protein content was determine according to the Kjeldahl’s method in GB5009.5-2016 “Determination of Protein in Food”; Ash content was determine based on GB5009.4-2016 “Determination of ash in food; Moisture content was determined by MB35 Ohaus moisture analyzer; SDF (soluble dietary fiber), IDF and TDF content were determined using GB5009.88-2014 “Determination of Dietary Fiber in Food”.

### 2.3. Structure of the Three Different Granularity of OIDFs

#### 2.3.1. Particle Size Determination

In total, a 0.1% (*m*/*v*) OIDF-10, OIDF-50 and OIDF-100 suspension was prepared. The particle size and specific surface area were analyzed using a BT-9300HT laser particle sizer (Bettersize Instruments Ltd., Dandong, China).

#### 2.3.2. Scanning Electron Microscopy (SEM)

After spraying with gold-palladium alloy, the microscopic appearance of OIDF-10, OIDF-50 and OIDF-100 was studied using a scanning electron microscope (SEM; Zeiss Supra55VP, Carl Zeiss, Jena, Germany). The scanning pictures were taken at magnifications ranging from 1000 (scale bar 20 µm) to 8000 (scale bar 2 µm) at accelerating voltages of 20 kV.

#### 2.3.3. Fourier Infrared Spectrum (FT-IR)

The three distinct granularities of OIDF were evenly mixed with KBr at a 1/100 (*w*/*w*) ratio and pressed into tablets. Each sample was scanned using a Fourier-infrared spectrometer (IRTracer-100, Shimadzu, Kyoto, Japan) throughout a wave range of 4000 cm^−1^ to 400 cm^−1^ with a resolution of 4 cm^−1^.

#### 2.3.4. Determination of Thermogravimetry (TG)

Thermogravimetry (TG) is a technical method to measure the relationship between the mass of a substance and temperature under the condition of a program-controlled specific temperature. Thermogravimetric analysis of OIDF-10, OIDF-50, and OIDF-100 was carried out by the HCT-3 microcomputer differential thermal balance. A sample of weighed 3–4 mg was placed into a crucible, placed on a sling balance, and the parameters were set as follows: initial temperature: 25 °C; heating rate: 10 °C/min; termination temperature: 600 °C; nitrogen flow: 50 mL/min. Mass and temperature/time were recorded continuously to obtain thermogravimetric curves.

#### 2.3.5. Determination of X-ray Diffraction (XRD)

The structures of OIDF-10, OIDF-50 and OIDF-100 were determined using an X-ray diffractometer model XRD-7000. Using Cu-Kα radiation (λ = 0.15418 nm), the working voltage of 40.0 kV, and a working current of 30.0 mA, we took an appropriate amount of sample and placed it on the surface of the sample plate, flattened it with a flat and smooth glass sheet, put it into the diffractometer, and set the parameters as follows: measuring angle: 5°~90°; step size: 0.02°; scanning speed: 4°/min. The specific crystallinity (degree of crystallinity) calculation formula is as follows:

(1) $$Dc(\%)=\frac{I_{002}-I_{am}}{I_{002}}\times 100$$

where *Dc*—degree of crystallinity; *I*_002_—diffraction intensity of crystalline region (2θ = 20.78°); *I_am_*—diffraction intensity of amorphous region (2θ = 18.0°).

### 2.4. Physicochemical Properties of the Three OIDFs

#### 2.4.1. Water-Holding Capacity (WHC), Oil-Holding Capacity (OHC) and Water-Swelling Capacity (WSC)

Referring to the method of Zhang et al. [20] with a slight modification, we accurately weighed 0.5 g of OIDF-10, OIDF-50 and OIDF-100 samples into 50 mL centrifuge tubes, added 30 mL of distilled water, shook them evenly and placed them at room temperature for 24 h. They were then centrifuged at 4000 r/min for 20 min, supernatant was then removed, and the mass of the precipitate was weighed. The calculation formula is as follows:

(2) $$\mathrm{WHC}(\mathrm{g/g})=\frac{W_{2}-W_{1}}{W_{1}}$$

where *W*_1_—sample mass (g); *W*_2_—precipitate mass after centrifugation (g).

We accurately weighed 0.5 g of OIDF-10, OIDF-50 and OIDF-100 samples in three separate 50 mL centrifuge tubes, added 30 mL of soybean oil, and evenly shook them. They were then centrifuged at 4000 r/min for 20 min, the upper layer oil was then removed, and the mass of the sediment was weighed. The calculation formula is as follows:

(3) $$\mathrm{OHC}(\mathrm{g/g})=\frac{W_{2}-W_{1}}{W_{1}}$$

where *W*_1_—sample mass (g); *W*_2_—precipitate mass after centrifugation (g).

We accurately weighed 0.5 g of OIDF-10, OIDF-50 and OIDF-100 samples in triplicate and placed them in a 25 mL graduated cylinder, and read the dry product volume V1. Then, we added 20 mL of distilled water to the graduated cylinder and mixed well. After standing at room temperature for 24 h, we recorded the volume of the fully swollen sample. The calculation formula is as follows:

(4) $$\mathrm{SW}(\mathrm{mL/g})=\frac{V_{2}-V_{1}}{W}$$

where *V*_1_—sample volume (mL); *V*_2_—swollen volume (mL); *W*—sample mass (g).

#### 2.4.2. Adsorption Capacity of the Three Particle Sizes of OIDF

##### Cholesterol-Adsorption Capacity (CAC)

A total of 1 mg/mL cholesterol in ethanol was made. Then, 0.2 g OIDF-10/OIDF-50/OIDF-100 was mixed with 10 mL cholesterol solution, adjusted to pH = 2/7 (adding water phase would precipitate cholesterol), incubated at 37 °C for 2 h (pH = 2/4) or 4 h (pH = 7), centrifuged at 4000 rpm for 20 min, and supernatant obtained to test cholesterol concentration using the OPA technique [21].

##### Sodium Cholate-Adsorption Capacity (SCAC)

A total of 0.1 g OIDF-10/OIDF-50/OIDF-100 was mixed with 10 mL sodium cholate standard solution (0.2 g sodium cholate + 15 mmol/L NaCl aq 100 mL), adjusted to pH = 2, 4, 7, incubated at 37 °C for 2 h (pH = 2, 4) or 4 h (pH = 7), centrifuged at 4000 rpm for 20 min, and the permeate was taken to determine the exact concentration of sodium cholate using this method [22].

### 2.5. Animals and Experimental Diets

The experiment was conducted in compliance with Jilin Agricultural University’s Laboratory Animals Guidelines and was authorized by Jilin Agricultural University’s Laboratory Animal Welfare and Ethics Committee (no. 20210422001). SD rats were kept in a temperature-controlled (20–25 °C) environment with a 12-h light/dark cycle. SD rats were randomly separated into five groups (*n* = 10) after a week of adaption, with the average weight in each group being comparable. The diet for each of the five groups of SD rats was divided into a normal diet (NC), HD, HD-OIDF-10, HD-OIDF-50, HD-OIDf-100. NC: Normal diet rats; HD: high-fat diet rats; HD-OIDF-10: High-fat diet + OIDF-10 (1000 mg/kg); HD-OIDF-50: High-fat diet + OIDF-50 (1000 mg/kg); HD-OIDF-100: High-fat diet + OIDF-100 (1000 mg/kg); After eight weeks of feeding, all rats were starved for 12 h and then anesthetized at the end of the experiment. For the serum preparation, blood samples were obtained and kept at −80 °C for future use. The liver and cecal contents were kept at a temperature of −80 °C. The composition of diets for each group is shown in Table 1.

### 2.6. Lipid Analysis

The blood concentrations of TC, TG, LDL-C, HDL-C, were measured using commercial test kits employing an enzymatic technique. A microplate reader was used to measure all of the data.

### 2.7. Histological Analysis

Liver tissues were fixed in four percent paraformaldehyde, embedded in paraffin for tissue slice preparation (5 m thickness), and stained with hematoxylin and eosin for histological investigation (H&E).

### 2.8. RNA Extraction and Quantitative Real-Time PCR Analysis

Total RNA was extracted from the frozen liver using Trizol reagent according to the manufacturer’s instructions for quantitative real-time PCR analysis. A spectrophotometer (Thermo Fisher Scientific, Waltham, MA, USA) was used to determine the RNA content and purity at 260 and 280 nm. The cDNA Synthesis Kit was used to reverse-transcribe the total RNA (1 g) into cDNA. Then, using a Biometra TProfessional PCR, mRNA expression was measured using quantitative real-time PCR with the SYBR Premix Ex Taq TM mix (Takara, Shiga, Japan). The manufacturers’ websites include the sequences of the primers that were used to amplify the target genes. The subsequent PCR amplification protocol was used: 95 °C for 30 s, followed by 40 cycles of 95 °C for 10 s, 56 °C for 30 s, and 72 °C for 30 s. The 2-ΔΔCT technique was used to calculate relative quantification.

### 2.9. Western Blot Analyses

RIPA lysis buffer combined with a protease inhibitor cocktail was used to lyse liver samples (Beyotime Biotechnology, Shanghai, China). The lysates were centrifuged for 20 min at 12,000 rpm at 4 °C, the supernatants were collected, and the protein concentration was measured using the BCA protein assay kit (Beyotime Biotechnology, Shanghai, China). SDS-PAGE was used to separate the protein lysates, which were then transferred to PVDF membranes. The blotted membranes were blocked for 2 h with 5% skimmed milk, then washed and incubated overnight at 4 °C with antibodies against CYP7A1, HMG-CoA, LDL-R. The bands were seen using an enhanced chemiluminescence reagent and assessed in an iBright CL1000 imaging system after incubation with the relevant primary and secondary antibodies (Invitrogen, Singapore). The expression of β-actin was used as a control for normalizing protein expressions.

### 2.10. Gut Microbiota Sequencing Analysis

For the sequencing of cecal contents for microflora sequencing, the QIAamp^®^ DNA Stool Mini Kit (QIAGEN, Hilden, Germany) was used to extract DNA from feces according to the manufacturer’s instructions. The quality of extracted DNAs was assessed using an agarose gel electrophoresis and subsequently a NanoDrop NC2000 spectrophotometer to precisely measure the DNA concentration (Thermo Fisher, Waltham, MA, USA).

Each sample’s pure DNA was used to amplify the V3–V4 regions of the 16S rRNA gene. With forward primers, 338F (5′-ACTCCTACGGGAGGCAGCA-3′) and the reverse primer 806R (5′-GGACTACHVGGGTWTCTAAT-3′) were utilized in the PCR amplification. The Data science PicoGreen dsDNA Assay Kit was used to determine the concentrations of PCR products (Invitrogen, Carlsbad, CA, USA). Following that, all of the PCR results were pooled in equal proportions, and paired-end sequencing was conducted using the Illumina MiSeq platform, which is run by Shanghai Personal Biotechnology Co., Ltd. (Shanghai, China).

### 2.11. Statistical Analysis

The trials were conducted three times, and the findings were presented as mean, standard deviations (SD). GraphPad Prism 6 was used for statistical analysis (GraphPad Software, Inc., California, CA, USA). Using IBM SPSS 25.0 (SPSS Inc., Chicago, IL, USA), were evaluated using one-way analysis of variance (ANOVA), and *p* < 0.05 was considered to be statistically significant.

## 3. Results

### 3.1. Determination of the Basic Components of the Three OIDFs

As shown in Table 2, the protein level of OIDF reduced from 1.85% to 1.63% after it was digested by DHPM. The TDF content of OIDF-10 increased, as did the TDF and SDF (soluble dietary fiber) content, which could be attributed to the fact that after high-pressure treatment, some insoluble hemicellulose, cellulose, and pectin, etc., link bonds are broken, the structure is destroyed, and the molecules are converted into soluble short-chain small molecules. This could be owing to the DF’s intricate structure. DF and protein particles will agglomerate to form a whole in most cases [23]. They are subjected to various strong effects such as pressure, shear force, and friction force during the high-pressure microfluidic process, resulting in part of the protein being converted into a free state and released, so it gradually increases with the increase in pressure. During the particle size reduction process, the gliadin protein is lost, and the protein content is reduced.

### 3.2. Structure of the Three Different Granularity of OIDFs

**Figure 1:**
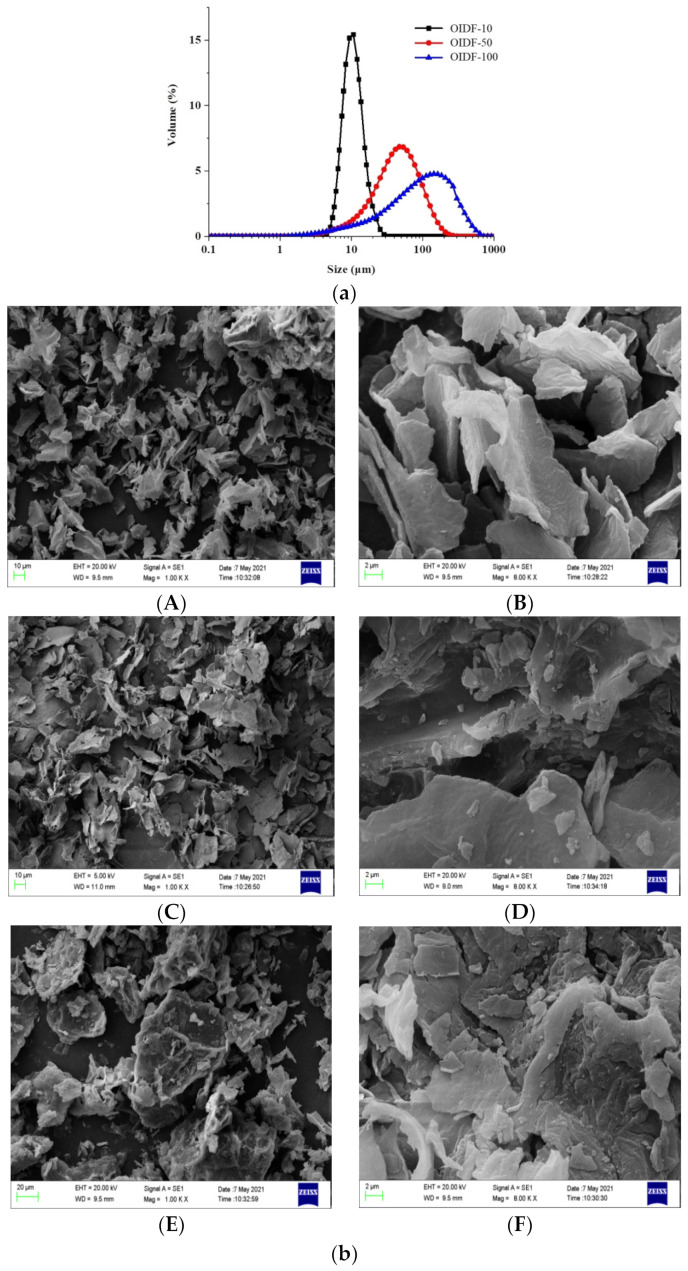

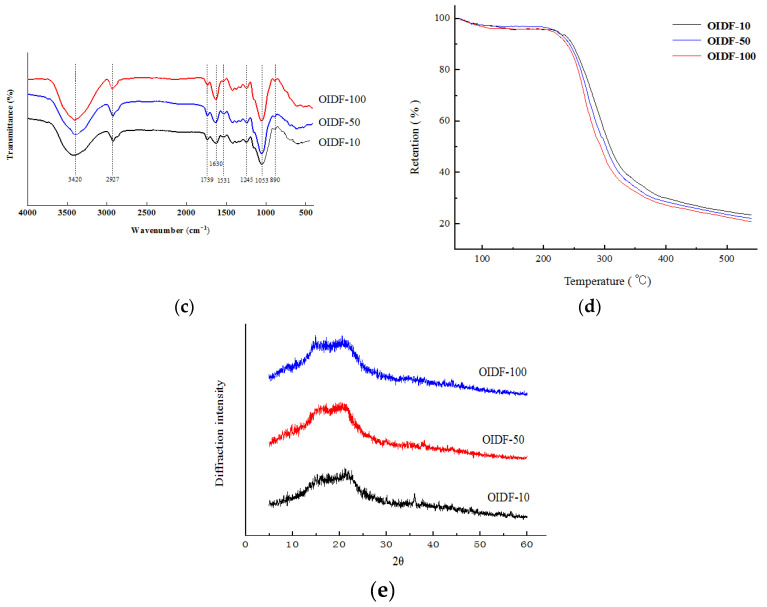
Structural characterization of OIDF with three particle sizes: (**a**) Particle size characterization; (**b**) SEM characterization; (**c**) FI-IR characterization; (**d**) TG characterization; (**e**) XRD characterization). Figure 1b (**A**: OIDF-10, 1000×; **B**: OIDF-10, 8000×; **C**: OIDF-50, 1000×; **D**: OIDF-50, 8000×; **E**: OIDF-100, 1000×; **F**: OIDF-100, 8000×).

From Figure 1a, with regard to particle size analysis: the particle size distribution curves of the three OIDFs were all unimodal, and the particle size distribution of OIDF-10 is relatively concentrated, with a higher peak and a normal distribution. When the average particle size of OIDF is smaller and more uniform, the difference in its physical and chemical properties is noticeable.

From Figure 1b, as shown in the SEM figure, the surface of OIDF-100 processed by 10 MPa pressure processing is relatively smooth, the structure is compact and complete, but the particle size is not uniform, and there are large flake particles. The surface of OIDF-50 after high-pressure treatment at 50 MPa is rough, curled in many places, and the structure is partially damaged, which is related to the uneven force during grinding [24], The surface of the sample OIDF-10 is very different from OIDF-50 and OIDF-100, the particle size is significantly reduced, the surface is flocculent, becomes loose and porous, the internal structure is exposed, and there is a uniform accumulation phenomenon.

From Figure 1c, the FT-IR spectrum shows that the three particle sizes of OIDF have similar characteristic curve peaks, the broad peak at 3420 cm^−1^ is the stretching vibration peak of hydroxyl (-OH), and hydrogen bonds in cellulose and hemicellulose [25] are a characteristic band of all cellulose. The absorption peak at 2927 cm^−1^ is the stretching vibration peak on the carboxymethyl group and methylene group (-CH) in hemicellulose [26]. Characteristic absorption peaks at 1739 cm^−1^ and 1630 cm^−1^ are due to the asymmetric stretching vibrations of C=O of acetyl groups or esters, including methyl esterification or free carboxyl groups [27]. As the particle size decreases, the intensity of the absorption peak gradually weakens, which may be due to the destruction of some ester structures during the grinding process. There is also literature showing that the weakening of the absorption peak here will lead to a poor water holding capacity [28], which provides a theory for water holding research. The absorption peak at 1531 cm^−1^ is the characteristic absorption peak of C=O in the imide bond; the absorption peak at 1245 cm^−1^ is the vibration of the OH or -CO group of hemicellulose, and 1053 cm^−1^ is the C-O-C stretching vibration peak. The absorption peak at 890 cm^−1^ is the characteristic absorption peak of the β-configuration glycosidic bond [29]. To sum up, compared with the characteristic peaks of OIDF-50 and OIDF-100, the peak shape, position and number of OIDF-10 did not change significantly, indicating that the main components and structure of OIDF did not change substantially after DHPM treatment.

From Figure 1d, the OIDF thermogravimetric curves of the three particle sizes showed the same trend, mainly composed of four stages. The first stage is the drying stage. From the initial temperature of 25 °C to 110.25 °C, the thermogravimetric curves of the three particle sizes of OIDF all show a weight loss of about 3.9%, which is due to the evaporation of free water and crystal water inside the fiber. The second stage is the pre-carbonization stage, the temperature range is 110.25–208 °C, and the weight loss is 0.73%, which belongs to the process of slow decomposition of macromolecules; the third stage occurs at about 208.38–355.55 °C, which belongs to the carbonization stage. The decrease was significant, with weight loss as high as 62%, which may be due to thermal decomposition or degradation of cellulose and hemicellulose [30]. It is consistent with the conclusion obtained by Fourier transform infrared spectroscopic analysis of OIDF with different particle sizes. The final stage is the combustion stage. When the temperature exceeds 355 °C, the thermogravimetric curve trend is stable due to the gradual decomposition of the residual sample into carbon and ash. The retention rate is the percentage of the final residue of the sample to the original mass. The retention rate of OIDF-10 is 22.89%, the retention rate of OIDF-50 is 21.77%, and the retention rate of OIDF-100 is 20.35%.

As shown in Figure 1e, any crystalline substance has its own unique X-ray diffraction pattern, and the position and shape of the characteristic diffraction peaks can be used to qualitatively and quantitatively analyze the substance, and to determine the crystal structure and degree of crystallinity. The results are shown in Figure 1e. The diffraction angles (2θ angles) are 15.42°, 20.78°, and 36.08°, which are typical characteristic diffraction peaks of cellulose, indicating that the crystal types of the three OIDFs belong to the cellulose I type. It is a state in which two phases coexist in a crystalline region and an amorphous region [31]. According to the calculation, the crystallinity of OIDF-100 is 33.65%, the crystallinity of OIDF-50 is 30.81%, and the crystallinity of OIDF-10 is 27.08%, indicating that the crystallinity of cellulose in OIDF-10 is significantly reduced after high-pressure microfluidization. Ullah et al. [32] also found that insoluble dietary fiber in soybean dregs was treated with high-energy wet media, and the crystallinity of cellulose gradually decreased with the prolongation of treatment time. In addition, Kang and Lu et al. [33,34] also came to the same conclusion.

### 3.3. Physicochemical Properties of the Three OIDFs

**Figure 2:**
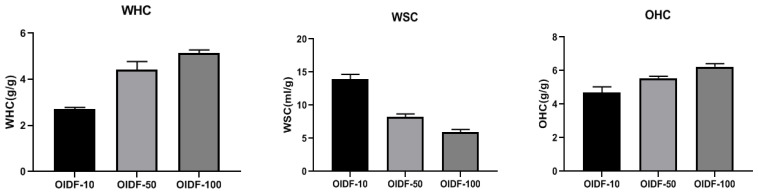
Effects of different particle sizes of OIDF on the water holding capacity (WHC), swelling capacity (WSC) and oil holding capacity (OHC).

The shear force is increased in the high-pressure micro-jet process, as illustrated in Figure 2, due to the mechanical effect of ultra-high pressure. Water holding capacity drops, oil holding capacity decreases, and swelling property rises when the OIDF particle size reduces, which might be related to an increase in the specific surface area as the particle size lowers. Ultra-high pressure damages the fiber structure, causing more hydroxyl groups of hydrophilic groups to be destroyed, resulting in a weakening of the fiber’s hydrophilic ability and a loss in water holding capacity and moisture content [35]. For example, Jasim et al. [36] and Zheng et al. [37] discovered that following ultrafine pulverization, the water-holding and oil-holding capabilities of insoluble dietary fiber declined as the particle size dropped. This might be due to certain hydroxyl and ester structures being destroyed during processing, which is consistent with the findings of this study.

**Figure 3:**
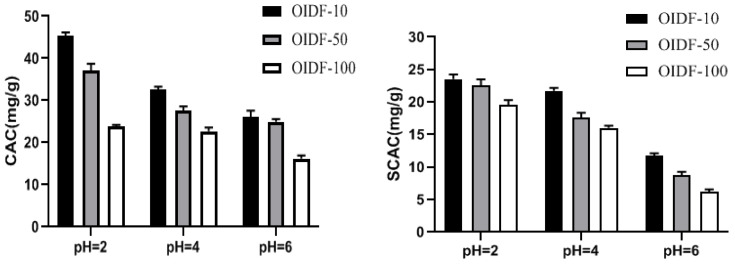
The effect of different particle sizes of OIDF on the adsorption of cholesterol (CAC) and sodium cholate (SCAC).

As shown in Figure 3, the three particle sizes of dietary fibers have varied adsorption capabilities for cholesterol in the three pH conditions. The smaller the particle size of dietary fibers in the same pH environment, the greater the adsorption capability. The adsorption ability of three types of dietary fiber to cholesterol steadily declined as pH increased. This finding suggests that dietary fiber has good cholesterol adsorption capabilities in a stomach acid environment. Similarly, in three different acidic circumstances, dietary fiber exhibited a similar adsorption rule to sodium cholate and adsorbed cholesterol, indicating that dietary fiber had high adsorption qualities to cholate in the stomach acid environment.

### 3.4. Effects of Different Particle Sizes of OIDF on Blood Lipid Levels in High-Fat Diet Rats

**Figure 4:**
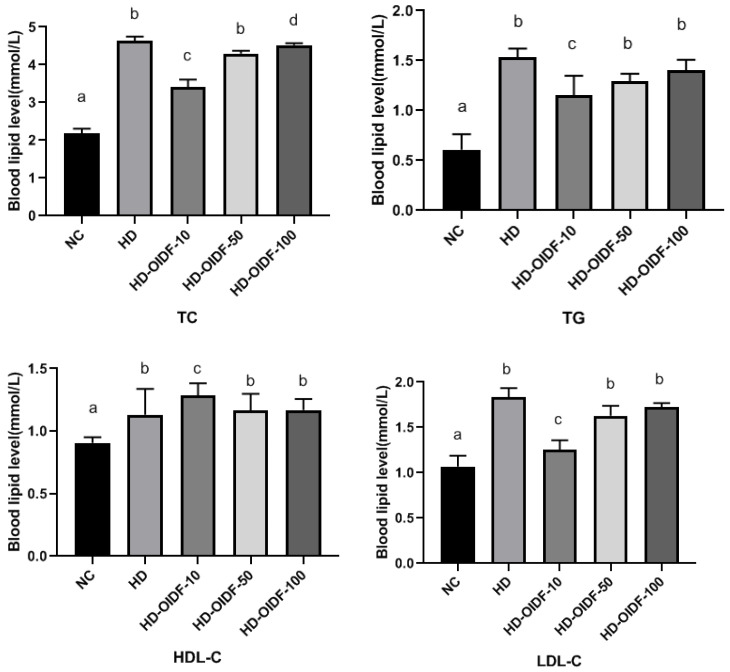
Effects of different particle sizes of OIDF on blood lipid levels in rats (TC, TG, HDL-C, LDL-C). Different letters in the same column (a, b, c and d) are significantly different (*p* < 0.05). The results are expressed as mean ± SD (*n* = 3).

As shown in Figure 4, a high-fat diet can significantly increase the content of TC and TG in blood. After OIDF intervention, there is a relief, and the group fed with OIDF-10 is the most significant. OIDF-50 and OIDF-100 groups have significant differences in reducing TC levels but no significant difference in reducing TG levels. This may be due to the large particle size, which leads to low intestinal utilization of OIDF, and thus, the reducing TG levels are not apparent. Regarding HDL-C, the OIDF-10 group significantly increased. HDL-C is mainly synthesized in the liver, which can promote the reverse transport of cholesterol, thereby achieving a lipid-lowering effect. Under a high-quality diet, the content of LDL-C in serum is higher, indicating that it is positively correlated with high blood lipids. OIDF-10 can significantly reduce LDL-C content, while the intervention effect of OIDF-50 and OIDF-100 is not significant. This is consistent with the findings of Zhou [38] and Hoang [39], but the exact reason is not clear.

### 3.5. Effects of Different Particle Sizes of OIDF on Hepatic Steatosis in High-Fat Diet Rats

**Figure 5:**
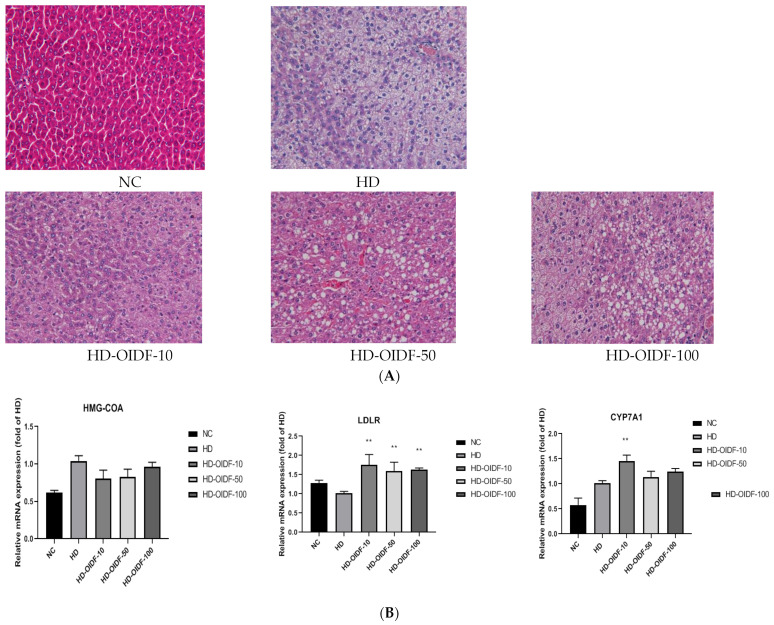

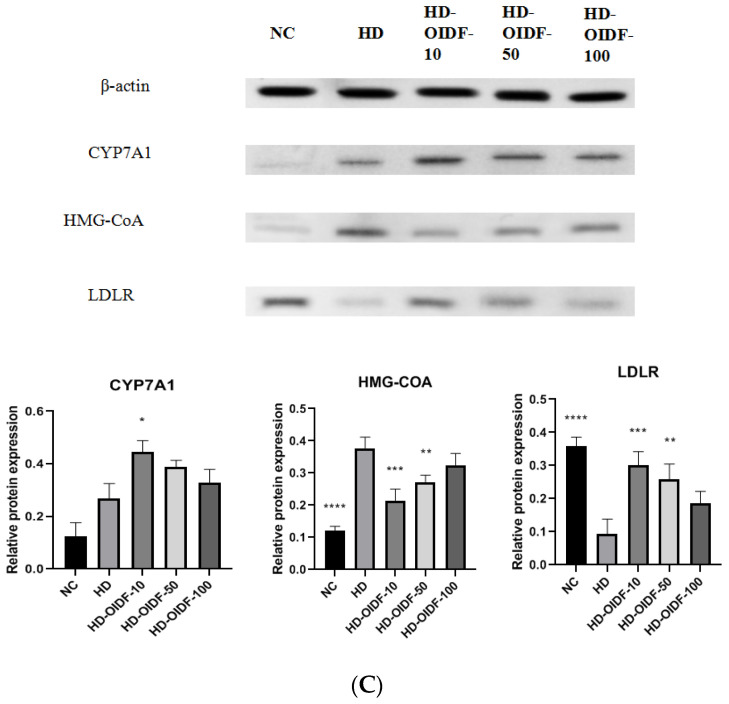
Effects of different particle sizes of OIDF on diet-induced hepatic steatosis in mice. Histological changes in liver sections were measured by H&E staining at 400× magnification: (**A**) Relative mRNA expression levels of CYP7A1, HMG-CoAr, LDL-R were determined by qRT-PCR; (**B**) The protein expressions of CYP7A1, HMG-CoAr and LDL-R in liver were determined by Western blotting; (**C**) Values are expressed as mean ± SD (*n* = 10). * represents *p* < 0.05, ** represents *p* < 0.01, and *** represents *p* < 0.001, and **** represents *p* < 0.0001, compared to the HD group. NC, normal diet-fed group; HD, high-fat diet-fed group; HD-OIDF-10, HD-OIDF-50, HD-OIDF-100, representing three distinct particle sizes of OIDF.

After H&E staining, the morphological changes of hepatocytes and fat droplets can be visually observed by microscope observation. The morphological observation results of the liver tissue of rats in each group are shown in Figure 5A. It can be seen from the figure that the size of hepatocytes in the NC group was normal throughout the eight weeks of feeding, the cells had complete cytoplasm and obvious nuclei, and the cell boundaries were clear and neatly arranged, while in the other groups, different degrees of accumulation of fat droplets were observed. In histological section, the white circles indicate fat droplets, which may be due to the accumulation of lipids in the body due to a long-term high-fat diet and the lack of exercise in cage feeding. After circulation in the liver, aggregation occurred. Different degrees of fat droplets were observed in other groups. Fat droplets accumulate. From the figure, we can clearly observe that in the HD group, after eight weeks of high-fat feeding, a large number of fat droplets appeared in the liver slices, and the liver was severely fatty. After eight weeks of intervention, compared with the HD group, the steatosis of liver cells in the HD-OIDF-50 and HD-OIDF-100 groups was slightly reduced, and the degree of lipid accumulation was not as fast as that in the HD group, but the morphological changes were different from those in the HD group, although not by much as the effect of inhibiting liver adipose is not as significant as the OIDF-10 group.

As shown in Figure 5B, RT-PCR was performed on the mRNA levels of three key genes of 3-hydroxy-3-methylglutaryl coenzyme (HMG-CoAr), Cholesterol 7α-hydroxylase (CYP7A1), and Low-density lipoprotein receptor (LDL-R) in rat liver. HMG-CoAr is a key rate-limiting enzyme for cholesterol synthesis. From the mRNA expression level of the gene, the relative expression of HMG-CoAr gene in the HD group was up-regulated in the NC group, indicating that a high-fat diet would increase the synthesis of cholesterol. This result was consistent with the results of lipid expression in serum and liver of HD group. As shown in Figure 5B,C, the effect of PCR was not significant. Combined with the analysis of the translation level Western Blot data, high-fat feeding in the HD group led to the high expression of HMG-CoAr gene, and OIDF intervention could reduce its expression, of which OIDF-10 had the most reduction effect. LDL-R can mediate the endocytosis of LDL and reduce the synthesis of hepatic cholesterol by increasing the reabsorption of LDL [40,41]. Therefore, the up-regulation of LDL-R in the OIDF-10 group may be due to the decrease in the blood lipid level of LDL-C. After eight weeks of OIDF dietary intervention, the expression level of CYP7A1 gene in the HD-OIDF-10 group was significantly higher than that in the HD group. Some researchers believe that FXR can activate bile acid synthesis by inducing the expression of CYP7A1 [42]. The high expression of the gene may promote the synthesis of bile acids, thereby promoting the metabolism of cholesterol.

The above three genes were confirmed by Western Blot, as shown in Figure 5C, and their protein level expression and mRNA expression trend were the same.

### 3.6. Bacterial Composition Analysis between Different Populations

**Figure 6:**
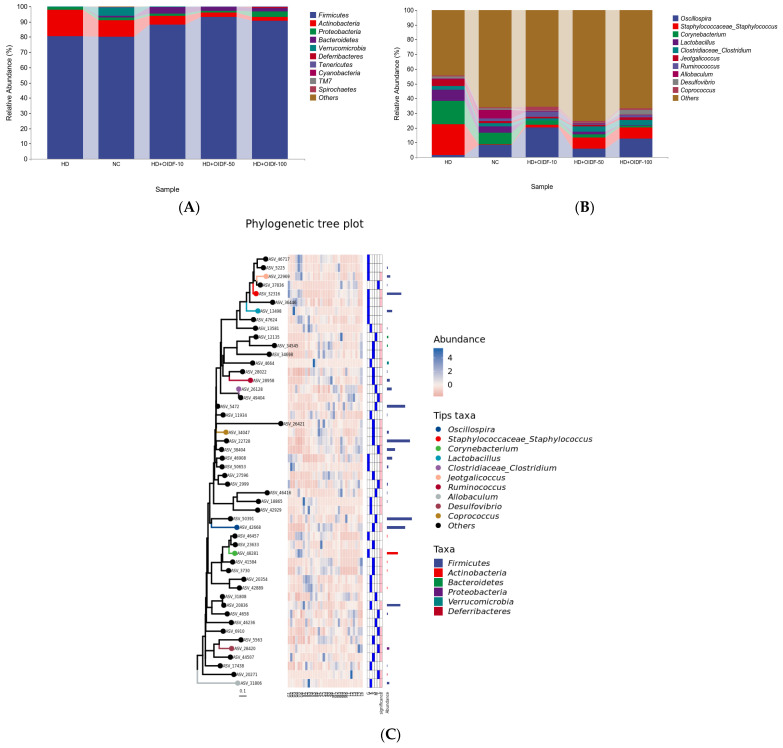
Bacterial composition analysis between different populations: (**A**) t phylum level; (**B**) genus level; (**C**) Species composition analysis.

By statistic on the ASV/OTU table after leveling, the specific composition table of the microbial community in each sample at each classification level can be obtained. Through this table, the number of taxa contained in different samples at each taxonomic level can be calculated first. Analysis software: QIIME2 (2019.4); Personal company self-compiled perl script. Analysis step: According to the results of the sequence species taxonomic annotation and the selected samples, we can count the number of taxa contained in each of the seven taxonomic levels of domain, phylum, class, order, family, genus, and species in the species annotation results of these samples. Some studies have used Firmicutes/Bacteroidetes ratios to explore the degree of obesity [43]. Compared with the HD group, the HD-OIDF-10 group was able to significantly reduce the ratio of Firmicutes/Bacteroidetes. As shown in Figure 6A and Table 3 (percentages shown in Table 3 are the mean value), at the phylum level, Firmicutes NC group accounted for 80.32%, HD group accounted for 80.67%, HD-OIDF-10 group accounted for 88.25%, HD-OIDF-50 group accounted for 93.04%, HD-OIDF-100 group accounted for 90.64%. Firmicutes are the dominant beneficial bacteria in the intestinal tract, indicating that OIDF with different particle sizes can increase the abundance of the Firmicutes after the intervention of a high-fat diet. Regarding Bacteroidetes, the NC group accounted for 1.27%, the HD group 0.03%, the HD-OIDF-10 group 4.44%, the HD-OIDF-50 group 2.34%, and the HD-OIDF-100 group 2.06%. Bacteroidetes are engaged in colonic metabolism, including glucose fermentation and bile acid and steroid biotransformation, and their numbers have grown. In the HD group, a high-fat diet resulted in a significant reduction in Bacteroidetes. The proportion of Bacteroidetes rose after OIDF intervention, notably in the HD-OIDF-10 group. The OIDF-10 intervention might dramatically reduce the rise in *Actinobacteria* and *Proteobacteria* produced by a high-fat diet when compared to the NC group. At the genus level, as shown in Figure 6B, Oscillospira showed the largest quantitative difference among the groups, and Oscillospira was able to produce short-chain fatty acids (SCFAs) such as butyrate, suggesting that it may play an essential role in various aspects of human function and health [44]. At the same time, studies have shown that Oscillospira can ferment complex plant carbohydrates [44]. After the intervention of three particle sizes of OIDF, the content of Oscillospira in the HD-OIDF-10 group was the highest at 20.25%, which was significantly higher than that in the HD group by 2.4 times. It shows that the bacteria in the gut can obtain carbon sources by decomposing the plant polysaccharide OIDF. As shown in Figure 6C, the elements of the phylogenetic tree graph mainly include: the phylogenetic tree graph, coloring ASV characteristic sequences or OTU representative sequences (tips in the graph) according to the taxonomic level. The first column is the sequence ID, and the second column is the relative abundance of the sequence in each sample of the grouping scheme. From left to right are the HD group, the NC group, the OIDF-10 group, the OIDF-50 group, and the OIDF-100 group, with six samples in each group. As shown in Table 3, at the genus level, OIDF was also able to modulate gut microbiota composition, enabling the development of gut microbiota towards a healthy state, reducing the abundance in *Allobaculum* bacteria in the gut of rats on a high-fat diet. *Ruminococcus*, ruminant digestive tract flora, is fermented to produce lactic acid, and the OIDF was able to increase *Ruminococcus* abundance, of which OIDF-10 had the most significant effect, possibly through the production of lactate, which is produced by gut bacteria, making a valuable contribution to colon health. It helps promote gut health and the bacteria that produce it protect against disease.

### 3.7. Alpha Diversity Index and Beta diversity PCoA Analysis

**Figure 7:**
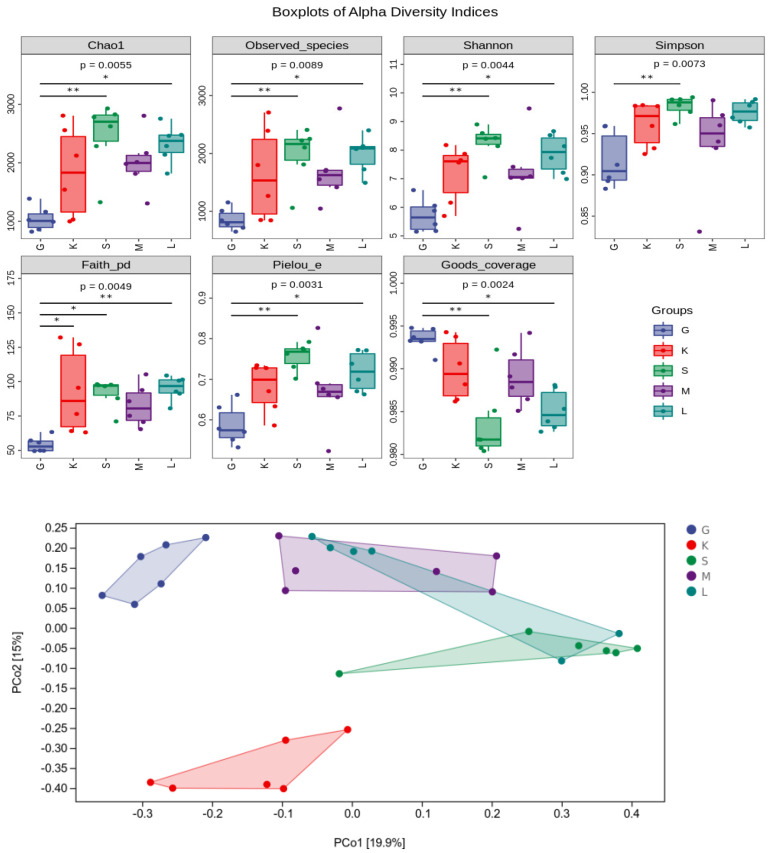
Alpha Diversity Index and Beta diversity PCoA analysis (G: HD group; K: NC group; S: HD-OIDF-10 group; M: HD-OIDF-50 group; L: HD-OIDF-100 group. * represents *p* < 0.05, ** represents *p* < 0.01).

As can be seen from Figure 7, the chao1 index of intestinal flora in the HD group was the smallest, which is a measure of its richness. The emphasis on rare species was the lowest, which was significantly different from the HD-OIDF-10 group. The Shannon index of intestinal flora in the HD group was significantly lower than that in the NC and HD-OIDF-10 groups (*p* < 0.01), that is, the intestinal microecological diversity of the HD group was significantly lower than that of the NC and OIDF intervention groups. The reason may be due to the high-fat diet changes the intestinal ecology, and the excessive reproduction of harmful bacteria that reduces the diversity of intestinal flora [40]. The richness and diversity of the intestinal flora of the HD-OIDF-10 group were closer to those of the NC group, and the results of other types of the analysis showed the same trend, indicating that the species richness between HD and NC, OIDF-10, and OIDF-100 and diversity are significantly different.

As the picture shows, β-diversity analysis can be used to study the similarity and difference of the bacterial community structure in different samples. Commonly used analysis methods include sample hierarchical cluster analysis, principal coordinate analysis, and multi-dimensional scaling analysis [45]. This study focuses on principal coordinate analysis (PCoA). As shown in Figure 7, the HD group and the NC group were clustered separately. The difference was obvious, that is, the microflora structure of the high-fat diet rats was different from that of the normal mice. In contrast, the coordinates of the OIDF dietary intervention group were shifted to the NC group, indicating that OIDF was able to modulate gut microbiota composition in rats fed a high-fat diet. Figure 7 shows that HD-OIDF-10 intervention in high-fat diet rats has a significant difference with HD group rats (*p* < 0.05), which indicates that OIDF-10 can change the intestinal flora structure of high-fat diet rats. These results suggest that OIDF can modulate the gut microbiota structure in rats fed a high-fat diet.

### 3.8. Alpha Diversity Index and Beta Diversity PCoA Analysis

**Figure 8:**
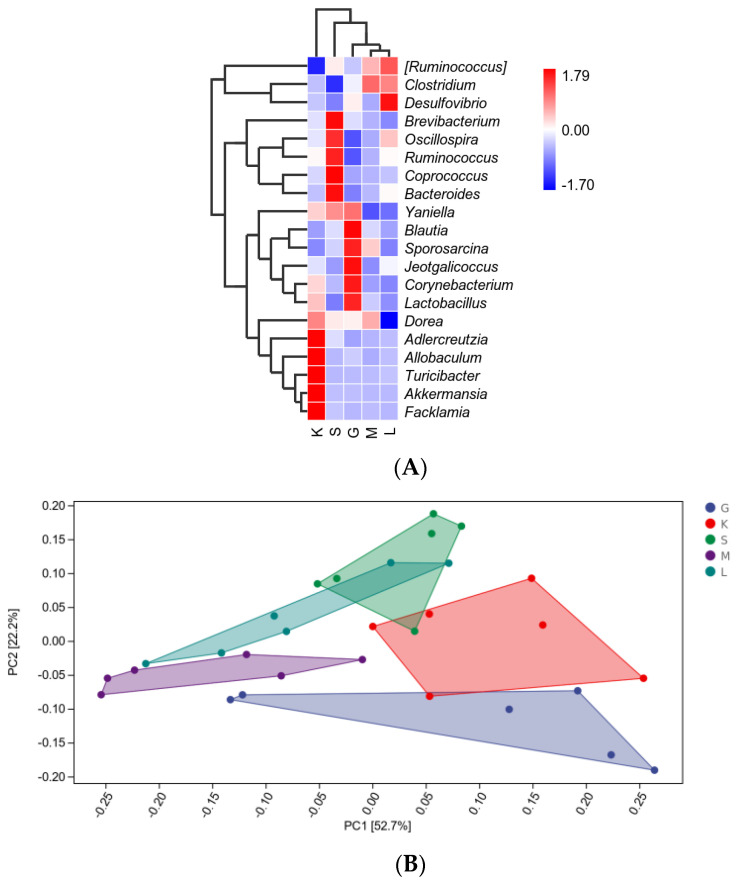

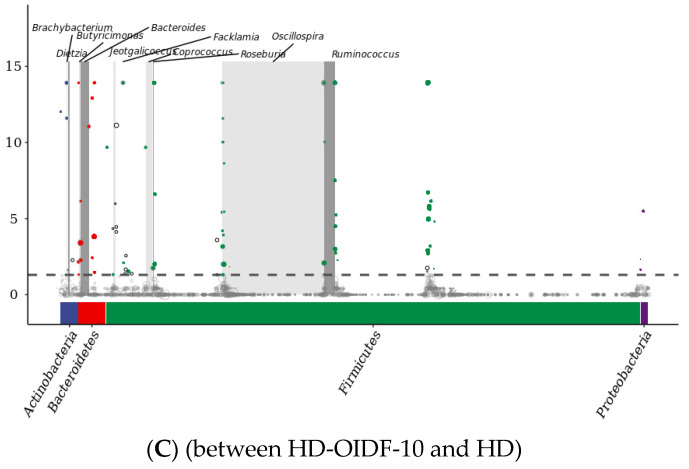
Species differences and marker analysis: (**A**) Species composition heatmap; (**B**): PCA analysis; (**C**): MetagenomeSeq analysis. (G: HD group; K: NC group; S: HD-OIDF-10 group; M: HD-OIDF-50 group; L: HD-OIDF-100 group).

As shown in Figure 8A, cluster analysis was performed between each group, and the top 20 bacterial species were obtained, which were *Ruminococcus*, *Clostridium*, *Desulfovibrio*, *Brevibacterium*, *Oscillospira*, *Ruminococcus*, *Coprococcus*, *Bacteroides*, *Yaniella*, *Blautia*, *Sporosarcina*, *Jeotgalicoccus*, *Corynebacterium*, *Lactobacillus*, *Dorea*, *Adlercreutzia*, *Allobaculum*, *Turicibacter*, *Akkermansia*, *Facklamia*. Compared with the HD group, HD-OIDF-10 group increased the abundance of *Brevibacterium*, *Oscillospira*, *Ruminococcus*, *Coprococcus*, *Bacteroides*, *Yaniella*, and HD-OIDF-50 increased the abundance of *Ruminococcus*, *Clostridium*, HD-OIDF-100 compared with HD group increased the abundance of *Ruminococcus*, *Clostridium*, *Desulfovibrio*, *Oscillospira*, *Ruminococcus*, *Bacteroides* compared with the HD group, indicating that the OIDF can improve the dysbiosis caused by a high-fat diet. As shown in Figure 8B, in the PCA analysis, PC1 = 52.7%, PC2 = 22.2%, and the differences in PCA analysis between groups were significant. The HD-OIDF-10 group and the HD group with extremely significant differences were selected for MetagenomeSeq analysis, and the occurrence frequency of ASV was greater than or equal to 0.3 as the condition, as shown in Figure 8C. The sample groups were compared using the metagenomeSeq method. This method avoids the influence of data sparse (Rarefaction) process on the accuracy of results, and is especially suitable for sparse microbial composition data. The dotted line separated the significant difference (above) and the insignificant ASV, significant difference. The points are marked by colored dots or circles, gray circles indicate the insignificant ones and the significantly up-regulated ones in this group are indicated by colored solid circles. The color of the dots identifies its phylum level name and is marked at the bottom of the figure; for the top 10 genera with significantly up-regulated points, a grayscale background is added, and the final analysis shows that the difference ASV number is 62, and after classification, it is concentrated in *Actinobacteria*, *Bacteroidetes*, *Firmicutes*, *Proteobacteria*.

## 4. Conclusions

In this study, three distinct particle sizes of OIDF were prepared from high-purity okara insoluble dietary fiber, and their structures and physicochemical properties were characterized, such as OIDF-10 having the higher WSC, OHC, CAC and SCAC. Using three distinct particle sizes of OIDF to interfere with the lipid-lowering effect of high-fat diet rats, we observed that OIDF supplementation could reduce blood lipid levels, alleviate hepatic steatosis, and regulate mRNA expression and protein of genes related to fat metabolism in the liver. The OIDF supplementation may provide a theoretical basis for OIDF with different particle sizes as functional food or dietary supplements by improving lipid metabolism and intestinal flora function in rats induced by a high-fat diet.

## Figures and Tables

**Table 1:** Composition of experimental diets.

Ingredient	NC		HFD/OIDF-10/OIDF-50/OIDF-100	
	gm	kcal	gm	kcal
Casein, 80 Mesh	200	800	200	800
L-Cystine	3	12	3	12
Corn Starch	315	1260	0	0
Maltodextrin 10	35	140	125	500
Sucrose	350	1400	68.8	275.2
Cellulose, BW200	50	0	50	0
Soybean Oil	25	225	25	225
Lard	20	180	245	2205
Mineral Mix	10	0	10	0
DiCalcium Phosphate	13	0	13	0
Calcium Carbonate	5.5	0	5.5	0
Potassium Citrate,1H_2_O	16.5	0	16.5	0
Vitamin Mix	10	40	10	40
Choline Bitartrate	2	0	2	0
FD&C Blue Dye#1	0.05	0	0.05	0
Total	1055.05	4057	773.85	4057
	Composition			
	gm%	kcal%	gm%	kcal%
Protein	19.2	20	26.2	20
Carbohydrate	67.3	70	26.3	20
Fat	4.3	10	34.9	60
Total		100		100
kcal/gm	3.85		5.24	

**Table 2:** Analysis of basic components of OIDF with different particle sizes (%).

OIDF	Protein (%)	Ash (%)	Moisture (%)	TDF (%)	IDF (%)	SDF (%)
OIDF-10	1.63 ± 0.28 ^a^	2.95 ± 0.11 ^a^	6.06 ± 0.56 ^a^	90.06 ± 0.12 ^a^	89.08 ± 0.04 ^a^	0.98 ± 0.09 ^a^
OIDF-50	1.66 ± 0.35 ^a^	3.05 ± 0.23 ^a^	5.87 ± 0.68 ^b^	89.42 ± 0.08 ^b^	88.63 ± 0.16 ^a^	0.79 ± 0.13 ^b^
OIDF-100	1.85 ± 0.24 ^b^	2.98 ± 0.18 ^a^	5.36 ± 0.84 ^c^	89.81 ± 0.06 ^b^	89.18 ± 0.03 ^a^	0.63 ± 0.11 ^c^

Different letters in the same column (a, b, c) are significantly different (*p* < 0.05). The results are expressed as mean ± SD (*n* = 3).

**Table 3:** Ratio of microbiota (top10) at the phylum and genus levels (%) (*n* = 6).

Taxonomy		NC	HD	HD-OIDF-10	HD-OIDF-50	HD-OIDF-100
phylum	*Firmicutes*	80.32%	80.67%	88.25%	93.04%	90.64%
	*Actinobacteria*	10.75%	17.04%	5.65%	2.94%	2.37%
	*Proteobacteria*	1.69%	2.06%	1.40%	1.15%	3.89%
	*Bacteroidetes*	1.27%	0.03%	4.44%	2.34%	2.06%
	*Verrucomicrobia*	5.40%	0.09%	0.10%	0.01%	0.01%
	*Deferribacteres*	0.01%	0.01%	0.05%	0.31%	0.81%
	*Tenericutes*	0.23%	0.02%	0.08%	0.12%	0.08%
	*Cyanobacteria*	0.04%	0.00%	0.00%	0.03%	0.01%
	*TM7*	0.02%	0.00%	0.01%	0.00%	0.02%
	*Spirochaetes*	0.01%	0.00%	0.00%	0.00%	0.00%
	*Others*	0.26%	0.08%	0.03%	0.07%	0.12%
Genus	*Oscillospira*	1.38%	8.40%	20.25%	5.71%	12.58%
	*Staphylococcaceae*	21.00%	0.47%	1.68%	7.87%	7.68%
	*Corynebacterium*	15.81%	7.72%	3.10%	2.08%	1.05%
	*Lactobacillus*	7.75%	4.22%	0.10%	1.77%	0.51%
	*Clostridiaceae*	2.51%	2.21%	1.27%	3.63%	3.43%
	*Jeotgalicoccus*	4.18%	1.67%	1.06%	0.95%	1.86%
	*Ruminococcus*	0.11%	1.65%	3.62%	0.91%	1.62%
	*Allobaculum*	0.58%	5.88%	0.26%	0.06%	0.37%
	*Desulfovibrio*	1.41%	0.85%	0.33%	0.64%	3.18%
	*Coprococcus*	0.74%	1.01%	2.69%	0.82%	0.90%
	*Others*	44.53%	65.91%	65.64%	75.57%	66.82%

## Data Availability

Raw data can be provided by the corresponding author on request.
